# 3D SERS Substrate of Z-Shaped Ag Nanorod Array for Thiabendazole Detection

**DOI:** 10.3390/molecules28207078

**Published:** 2023-10-13

**Authors:** Yongjun Zhang, Xiaoyu Zhao, Deyuan Mao, Jiahong Wen, Renxian Gao, Yaxin Wang

**Affiliations:** 1 School of Material and Environmental Engineering, Hangzhou Dianzi University, Hangzhou 310018, China; 2 Shangyu Institute of Science and Engineering, Hangzhou Dianzi University, Shaoxing 312000, China; wenjiahong@hdu.edu.cn; 3 The College of Electronics and Information, Hangzhou Dianzi University, Hangzhou 310018, China; 4 Department of Physics, Xiamen University, Xiamen 361005, China; renxiangao@xmu.edu.cn

**Keywords:** 3D hotspot distribution, Z-shaped Ag nanorods, thiabendazole detection

## Abstract

Ag nanoparticles sputtered on silicon wafer are used as masks for the fabrication of silicon columns by ion etching, which induces the growth of the inclined Ag nanorod by inclined Ag sputtering. V-shaped and Z-shaped Ag nanorods can be obtained by varying incline angles and deposition times. SERS detection and FDTD simulation are used to compare and investigate the enhanced electromagnetic coupling of incline nanorod arrays with different shapes in three-dimensional space, which indicates that Z-shaped nanorods show good SERS properties. The Z-shaped Ag nanorod array is used as a SERS substrate for the detection of thiabendazole with a concentration down to 10^−11^ M.

## 1. Introduction

Fungicides are usually used in fruits and vegetables to prevent mold, rot, and wilt, while in most cases the pesticide residue can pose hazards to public health and the environment [1,2,3]. Many pesticides remain in plant tissue for a long time after being applied to crops and fruits, which remain harmful to people because they have mutagenicity, cytotoxicity, and even carcinogenicity [4,5]. Thibendazole is also used to keep citrus fruits fresh after harvest, for example, bananas, apples, and pears. Because thiabendazole can cause thyroid hormone imbalance and liver damage, reliable and simple testing methods are urgently needed to prevent possible harm [6].

In food safety analysis, the standard method for analyte detection is based on chromatographic “wet chemistry” techniques such as gas chromatography (GC), high-performance liquid chromatography (HPLC), and mass spectrometry (MS). The main limitations of these methods include the need for expensive systems, expensive maintenance costs, and limitations in dedicated laboratory space, as well as the need for well-trained operators. LC–MS/MS analysis is usually the standard recommended for residue extraction due to its high molecular specificity and detection sensitivity, but the chromatographic techniques show inconvenient shortcomings, such as being time consuming, requiring large amounts of reagents, and high cost [7,8]. In recent years, a technique based on surface plasmon resonance (SPR) has attracted extensive attention for its wide applications.

The SPR phenomenon was discovered by Wood as early as 1902. When electromagnetic waves were directed towards metal surfaces, their reflection spectra would exhibit anomalies, manifested as a significant decrease in reflected light intensity at specific angles and the appearance of obvious dark bands in the spectra. Moreover, the increase in the refractive index of the metal film surface leads to a change in the position of dark bands. In 1941, the scientist Fano provided a reasonable explanation for the SPR phenomenon, indicating that the SPR phenomenon originates from the resonance of evanescent waves and metal surface plasma waves. Due to the presence of free electron gas in metals, the incident light stimulates the longitudinal vibration of the electron gas, and the charge density waves generated by the vibration propagate along the interface between the metal and the dielectric, forming surface plasma waves. When the metal nanostructures are excited by the incident light, local surface plasmon resonance (LSPR) occurs, exhibiting the strong absorption of light in a certain band and the significant enhancement of the electromagnetic field. Precious metal nanostructures such as Au, Ag, and Cu exhibit strong LSPR effects and strong spectral absorptions in the ultraviolet and visible light bands. The LSPR effect is the result of enhanced electromagnetic fields on the surface of precious metal nanoparticles, while SPR planar precious metals do not exhibit this effect. Due to the superiority of LSPR over SPR in these aspects, LSPR has replaced SPR in some trace detection applications. Precious metal nanoparticles, due to their unique optical properties, have strong plasmon resonance absorption bands that do not exist in the spectra of ordinary metals. At present, Au and Ag nanoparticles have received widespread attention and research in various nano optical applications, such as biochips and nanoscale applications. At the same time, LSPR-based devices can also be established simultaneously with simple optical systems, which makes the research on various sensors derived from LSPR-based precious metal nanoparticles very popular.

In 1998, Ebbesen et al. reported an abnormal optical transmission phenomenon on a metal nanopore array structure. The study showed that the incident light exceeded the diffraction limit and obtained an abnormal zero order transmission spectrum on a square metal array structure, which was attributed to surface plasmon resonance [9]. Since then, a lot of research has been carried out on surface plasmon excitation and optical properties for noble metal nanostructures. In 1999, Xu et al. proposed a preparation strategy of using sodium citrate to reduce silver nitrate and obtained aggregated Ag nanoparticles. Based on the LSPR effect between Ag nanoparticles, single molecule detection of oxygenated hemoglobin by Raman spectroscopy was achieved [10]. In 2003, Sun et al. reported the dependence of the LSPR coupling strength of two gold ellipsoid nanoparticles on the distance between the nanoparticles. The experiment showed that strong near-field interactions caused a red shift in the resonance wavelength of the LSPR. As the distance between nanoparticles decreased, the dependence was described by an exponential function at a certain scale [11]. Based on the characteristics of LSPR spectroscopy, plasmon structures have been applied to distance measurement at the nanoscale, namely nanorulers [12,13]. With the rapid development of plasmonics, some cutting-edge applications related to surface plasmons have been reported. The giant electromagnetic field enhancement effect can also be used for nonlinear optical processes. For example, in 2005, Muhlschlege et al. reported the polarization of the incident light along the two nanorod axes, in which the supercontinuum white light was generated [14]. In 2008, Eric et al. reported on the application of surface plasmons in color separation display and imaging. By designing textures on the surface of periodic metal array grooves at the nanoscale, the incident light was converted into surface plasmon waves, which separated them based on wavelength and polarization angle, finally obtaining spectra of different wavelengths again through the subwavelength aperture [15]. This was due to the physical effects of near-optical field enhancement, thermal energy, and the generation of hot electrons associated with LSPR, which is also the fundamental reason why plasmon structures are widely used in various fields.

As one important optical spectroscopy for chemical analysis, Raman spectroscopy is widely used for the detection, identification, and characterization of different compounds and biological species due to its molecular fingerprint effect, being suitable for sensing research such as in the field of food safety [16]. Raman spectroscopy is closely related to the oscillation and twisting of atoms and chemical bonds in matter, and the vibrational modes of different atomic clusters are unique, resulting in corresponding specific Raman spectra. Based on the fingerprint effect of Raman spectroscopy, Raman spectroscopy can be used for the analysis and identification of solid, liquid, gas, and other samples. Raman measurements have time-efficient properties but the main obstacle to traditional Raman is its low sensitivity due to limited scattering sections and efficiency. Due to the very weak Raman signal, it is difficult to analyze and detect the analytes in low concentrations, which limits the application of Raman spectroscopy [17]. When some target molecules are linked to or near the surfaces of the noble metal nanostructures, the local surface plasmon resonance from the metal nanostructures result in significant Raman peaks in comparison to the ordinary Raman spectrum, which is named as surface enhanced Raman scattering (SERS) [18,19,20]. In 1974, Fleischman et al. discovered that the Raman signal of pyridine probe molecules was greatly enhanced on rough Ag electrode surfaces, which was then explained as a surface effect [21]. Van Duyne and Albrecht et al. further confirmed the experimental phenomenon in 1977 and calculated an enhancement factor of approximately 10^5^–10^6^ [22,23]. Subsequently, Moskovits described that the enhancement of Raman signals originated from surface plasmons in metals, pioneering the connection between Raman spectroscopy and surface plasmons, thus opening up the door to the field of surface enhanced Raman scattering (SERS). After more than 40 years of development, SERS spectroscopy has gradually developed into a detection method with high sensitivity and strong spectral characteristics and is not easily affected by solutions. It has been widely applied in research fields such as chemistry, materials science, analytical science, surface science, biomedicine, and other important applications. Compared with traditional detection methods, SERS spectral detection technology has the advantages of ultra-high sensitivity for single molecule detection and can reflect the intrinsic fingerprint information of substances. In 2016, Radu et al. used SERS-based methods to simultaneously detect two types of vitamin B (riboflavin and cyanocobalamine) in vitamin-fortified grains [24]. The SERS-sensing platform has also been used to replace traditional chromatography techniques for monitoring and reliably detecting pesticide residues in food at trace levels. Surface sampling technology is commonly used for routine food screening to detect pesticides. Compared to fruit pulp, surface sampling technology has been found to be very effective in detecting pesticides on fruit skins. Chen et al. developed a SERS tape sensor decorated with gold glue particles for detecting pesticides in fresh agricultural products, demonstrating the practicality of surface sampling technology [25].

The characteristics of SERS active substrates are one of the key influencing factors affecting the development of SERS spectral detection technology. The stability and repeatability of SERS signals greatly depend on the material and structural characteristics of SERS active substrates. At present, the enhancement mechanisms of SERS can generally be divided into two types: physical enhancement and chemical enhancement. Physical enhancement refers to the enhanced observations due to electromagnetic coupling. Under the excitation of incident light, the vibration frequency of the electron cloud on the surface of the plasmon structure matches the vibration frequency of the excitation light, leading to a strong coupling resonance and localized light energy in a certain micro/nano space. This area is also known as the “hotspot” region. It is precisely due to the role of hotspots that Raman signals are greatly enhanced. In noble metal nanostructures, hotspots with high electromagnetic fields are created due to the significant coupling of the local surface plasmon resonance [26,27,28]. SERS gives us the opportunity to improve the detection limit to a single molecule level at these hotspots [29,30,31]. To improve observation, many efforts are devoted to design and prepare nano-array structures with more hotspots as SERS substrates [32]. The chemical enhancement mechanism mainly originates from the change in the polarization of probe molecules adsorbed on the surface of plasmon structures and is therefore interpreted as the charge transfer-enhancement mechanism. When excitation is applied to the plasmon structure, electrons will transition from their Fermi level to the adsorbed molecule or from the adsorbed molecule to the Fermi level of the substance, thereby altering the effective polarization of the probe molecule and enhancing the Raman signal to a certain extent. It is generally believed that the physical enhancement mechanism dominates, as the contribution of electromagnetic enhancement to SERS spectra is generally around 10^4^–10^6^, while the chemical charge transfer mechanism only contributes 10^2^–10^3^.

Although SERS suffers from some problems such as stability, repeatability, and high cost for analysis, SERS still attracts a lot of attention because of its unparalleled advantages such as minor susceptibility to aqueous environments, capability of in-situ detection, molecule-level sensitivity, and timely, accurate, nondestructive, and on-site observation [33,34]. The emerging three-dimensional SERS substrate exhibits excellent performance in sensing due to the addition of coupling models in a vertical direction, which expands the hotspot from the two-dimensional plane to the three-dimensional volume space [35,36,37]. The three-dimensional spatial SERS substrate provides extended parameters beyond the two-dimensional planar substrate to adjust plasma coupling in the vertical direction in three-dimensional space [38,39,40]. Due to the difficulty of fabricating three-dimensional spatial nanostructures in a controlled manner, the correlation between three-dimensional plasma coupling and SERS properties remains a challenge [41]. Although top-down lithography methods still face the challenge of preparing three-dimensional arrays of plasmonic metal nanoparticles, some work has shown how plasma coupling in three-dimensional space can improve SERS enhancement. Yüksel et al. reported that Ag or Au three-dimensional flower-shaped nanostructures can enhance hotspot density, further amplify SERS signals, and can be used as ultra-stable and reproducible SERS substrates [42]. Ye et al. reported Fe_3_O_4_@C@Ag 3D sea urchin-like nanostructures as SERS substrates can effectively detect organic pollutants in solutions [43]. Wang et al. reported that Fe_3_O_4_@SiO_2_@Ag nanostructures exhibit good signal enhancement [44]. Gao et al. prepared a uniform Ag nanowafer assembly film using copper plate as a support substrate, which has good reproducibility and sensitivity [45]. In this study, the inclined nanorod array was developed for the detection of thiabendazole. Silicon column substrates were obtained by plasma etching of silicon wafers, and inclined nanorods were grown by magnetron sputtering and inclined sputtering. V-shaped nanorods were obtained by inverting the substrate once, and Z-shaped nanorods were obtained by inverting the substrate twice. Z-shaped nanorods are confirmed as a substrate for thiabendazole detection.

## 2. Results and Discussion

**Figure 1:**
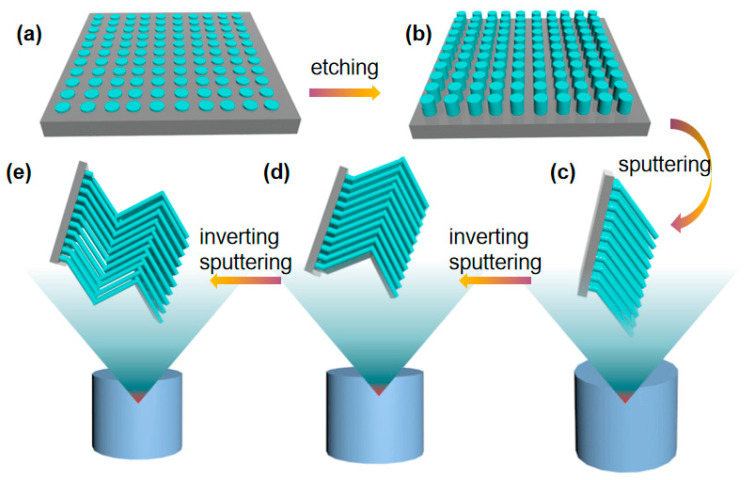
Illustration for the preparation of the V-shaped nanorods and the Z-shaped nanorods. (**a**) Sputtering metal nanoparticles on silicon wafers; (**b**) Reactive ion etching to get Si column; (**c**) Inclined growth of silver nanorods; (**d**) Growth of V-shaped silver nanorods obliquely with the substrate 180° rotation; (**e**) Growth of Z-shaped silver nanorods obliquely with second substrate 180° rotation.

Figure 1 illustrates the process for the fabrication of Ag nanorods. Firstly, Ag nanoparticles are sputtered onto Si substrate in a magnetron control sputtering system. Secondly, reactive ion etching is performed to get the silicon columns on the silicon wafer with Ag nanoparticles as the shielding. Thirdly, the tilted growth of Ag nanorods is induced by Si column with an inclination angle 70° to the horizontal plane. Finally, the V-shaped silver nanorods are obtained by rotating the substrate 180° for second deposition, and the Z-shaped nanorods are obtained for another 180° rotation and deposition.

**Figure 2:**
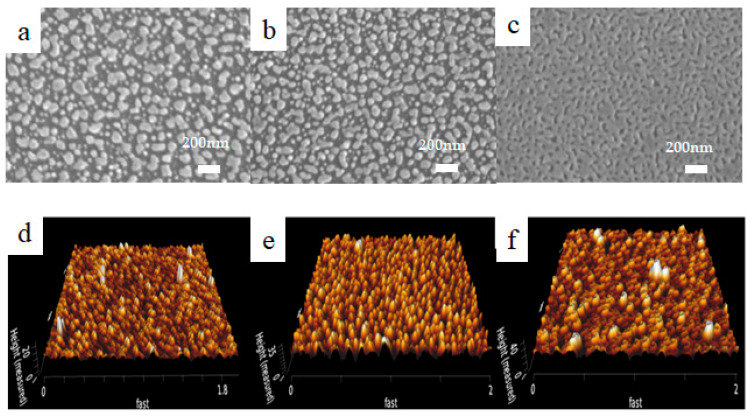
SEM and AFM images of samples etched for 20 s with sputtering Ag for (**a**,**d**) 20 s, (**b**,**e**) 40 s, and (**c**,**f**) 60 s.

When the silver nanoparticles are sputtered on the silicon wafer, silicon columns with different lateral sizes and densities are obtained, depending on the sputtering time for the silver nanoparticles. For sputtering time 20 s, 40 s, and 60 s, the silver nanoparticles work as masks during the etching process and the areas without silver nanoparticles will be etched away, which results in the significant silicon columns. In comparison to the sputtering time of 20 s, 40 s of sputtering leads to more silver nanoparticles with large densities, which results in the connected Si columns as shown in Figure 2a,b. When the sputtering time increases to 60 s, many pits can be seen in the silicon wafer, indicating the connection between Ag nanoparticles for long growth time. The corresponding atomic force microscopy (AFM) images also show that the nanoparticle distribution density increases with sputtering time and 40 s growth results in a uniform size distribution as compared to the sample growth for 20 s. For 60 s growth, the AFM image shows the obvious pit distributions, indicating the connected Si surface. According to the height scale, the heights of the silicon columns on the surface of the samples with the etching time of 40 s are 20 nm, 35 nm, and 40 nm, respectively.

**Figure 3:**
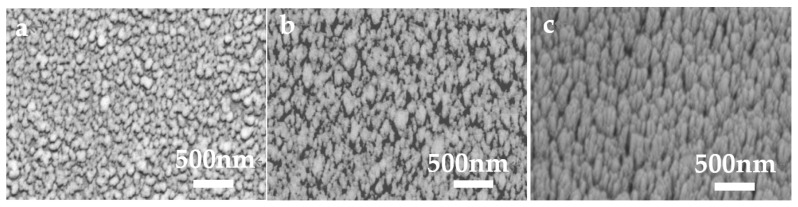
SEM images of Ag growth with different times, (**a**) 6 min; (**b**) 18 min; (**c**) 30 min.

**Figure 4:**
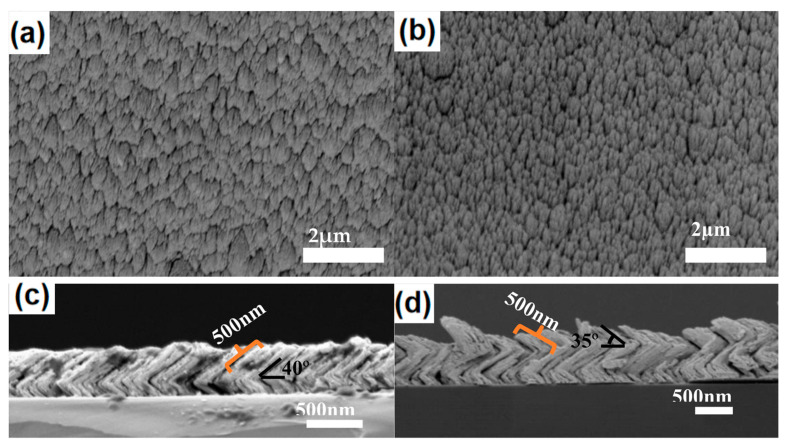
Top-view and cross-section view SEM images for the samples, (**a**,**c**) V-shaped nanorods; (**b**,**d**) Z-shaped nanorods.

As shown, 70° growth is performed by tilting the substrate, and the sputtering time is set as 6 min, 18 min, and 30 min respectively (Figure 3). SEM images show that some Ag nanoparticles were grown on Si columns. The Ag grows into the scattered and rough nanostructure instead of the continuous film, which indicates Ag growth is induced by the Si column. During Ag deposition, Si columns work as nuclei for Ag growth, which leads to the selective formation of Ag nanorods. Due to the shadow effect, no growth is found between Si columns, which leads to the formation of the scattered Ag nanostructures. Therefore, it is possible for the control of the lateral size of Ag nanorods by adjusting the density of the Si column. With the increased growth time, the oriented growth is obvious and the inclined Ag nanorods with lengths of about 500 nm can be obtained by the Ag sputtering time of 30 min. The inclination angle between the Ag nanorods and the plane of the Si wafer is measured as about 45°. To prepare Ag nanostructures with 3D hotspot distribution, V-shaped nanorods are grown by rotating the substrate 180°. By rotating the substrate 180° again, Z-shaped Ag nanorods are obtained, as shown in Figure 4.

The top-view images in Figure 4 show that the V-shaped and Z-shaped nanorods arrays are densely arranged with Ag nanorods. The tilted angle of the Z-shaped nanorods is small compared to the V-shaped. The section view images show that the diameter of the V-shaped and Z-shaped Ag nanorods is about 80 nm, and the gap between the silver nanorods is about 30 nm. The cross-sectional view of the V-shaped nanorods in Figure 4c shows that two segments of inclined nanorods are connected to each other for the V-shaped nanorods. The length of both segments of silver nanorods is 500 nm, and the inclination angle between the second segment of silver nanorods and the silicon wafer is around 40°. Figure 4d shows the section view of the Z-shaped nanorod, which shows that three inclined nanorods are connected to each other with a shape similar to the letter Z. The length of the three silver nanorods is 500 nm, and the inclination angle between the third silver nanorod and the plane of the silicon wafer is 35°.

**Figure 5:**
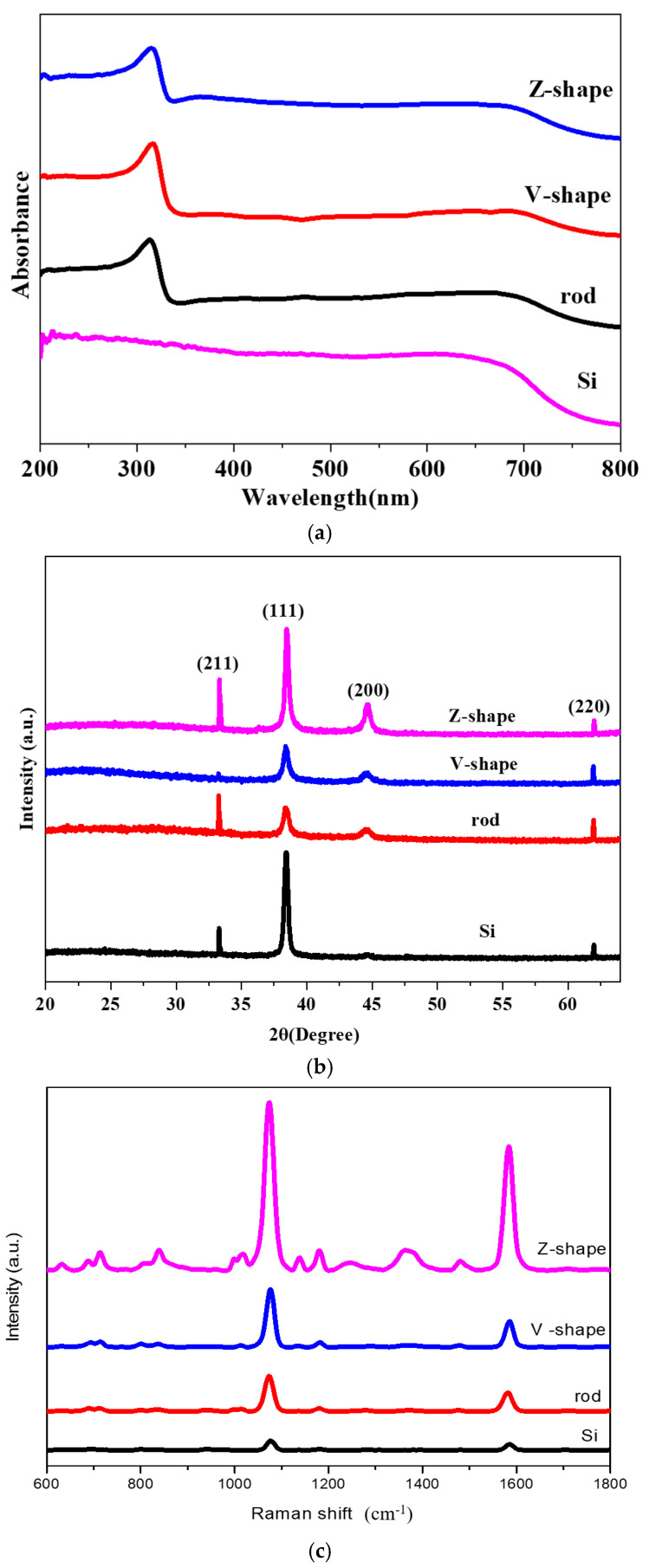
(**a**) UV absorption spectra of different nano array structures; (**b**) X-ray diffraction spectra of different nano array structures; (**c**) Raman spectroscopy of different nano array structures with 4-MBA as the probe molecule.

Figure 5a shows the absorption spectra of silicon wafers, tilted silver nanorod arrays, V-shaped nanorod arrays, and Z-shaped nanorod arrays. Compared to silicon wafers, the sample grown with silver nanorods has a clear absorption peak of around 300 nm. As the structure of silver nanorods becomes more complex, the absorption peak shows a significant red shift. Figure 5b shows the X-ray diffraction patterns of sputtered silver silicon wafers, tilted silver nanorod arrays, V-shaped nanorod arrays, and Z-shaped nanorod arrays, respectively. The peaks at 38° and 44° correspond to Ag (111) and Ag (200) and the peak at 33° corresponds to Si (211) of silicon. Using the silicon wafer sputtered with silver nanoparticles as the control, Raman detections were carried out respectively, so the average value of the sample was taken for five times of detection, so the sample successfully detected the Raman signal of the probe molecule. Figure 5c shows Ag nanoparticles on Si substrate gives the lowest Raman signal intensity, the Z-shaped nanorod array has the highest Raman signal intensity. The V-shaped nanorod array has a higher Raman signal intensity than the tilted nanorod array.

**Figure 6:**
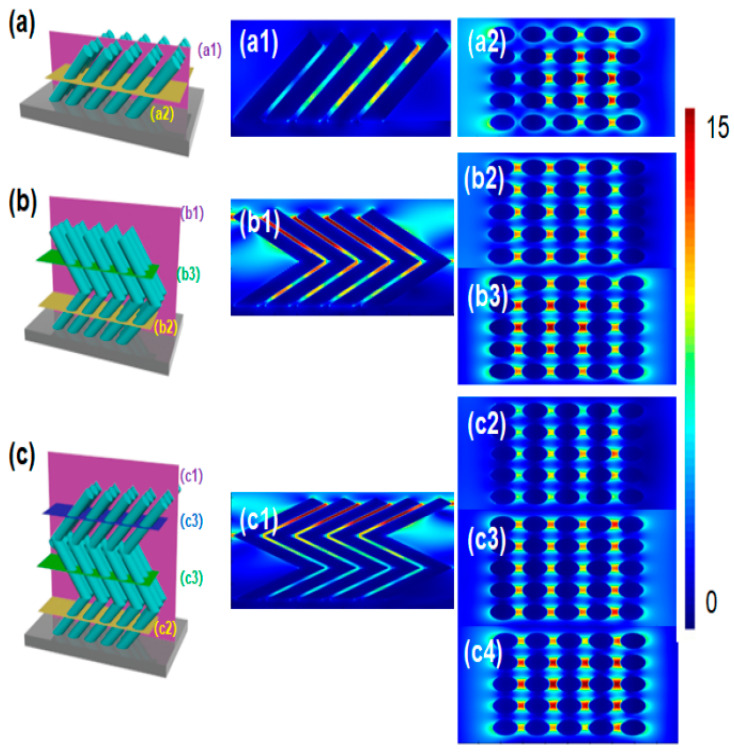
FDTD simulation models and electromagnetic coupling intensity distribution for different Ag nanostructures. (**a**) Tilted silver nanorod array, (**a1**) purple section position x = 0, (**a2**) yellow section position z = 176 nm; (**b**) V-shaped silver nanorod array simulation model, (**b1**) purple section position x = 0, (**b2**) yellow section position z = 176 nm, (**b3**) green section position z = 529 nm, (**c**) Z-shaped silver nanorod array simulation model, (**c1**) purple section position x = 0, (**c2**) yellow section position z = 176 nm, (**c3**) green section position z = 529 nm, (**c4**) blue section position z = 882 nm.

**Figure 7:**
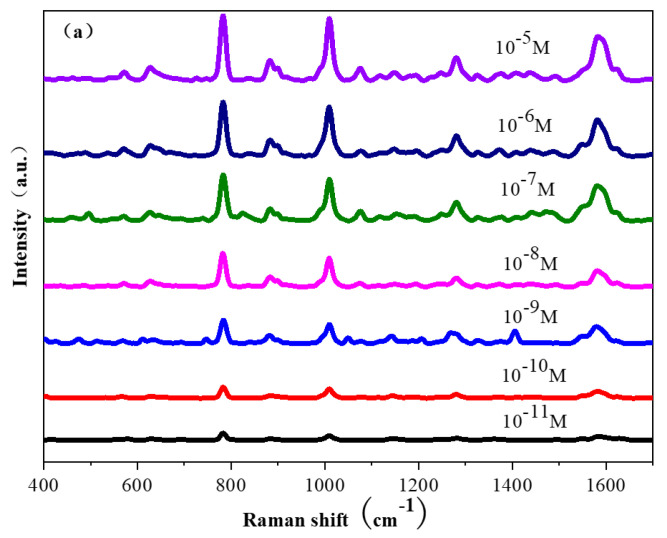

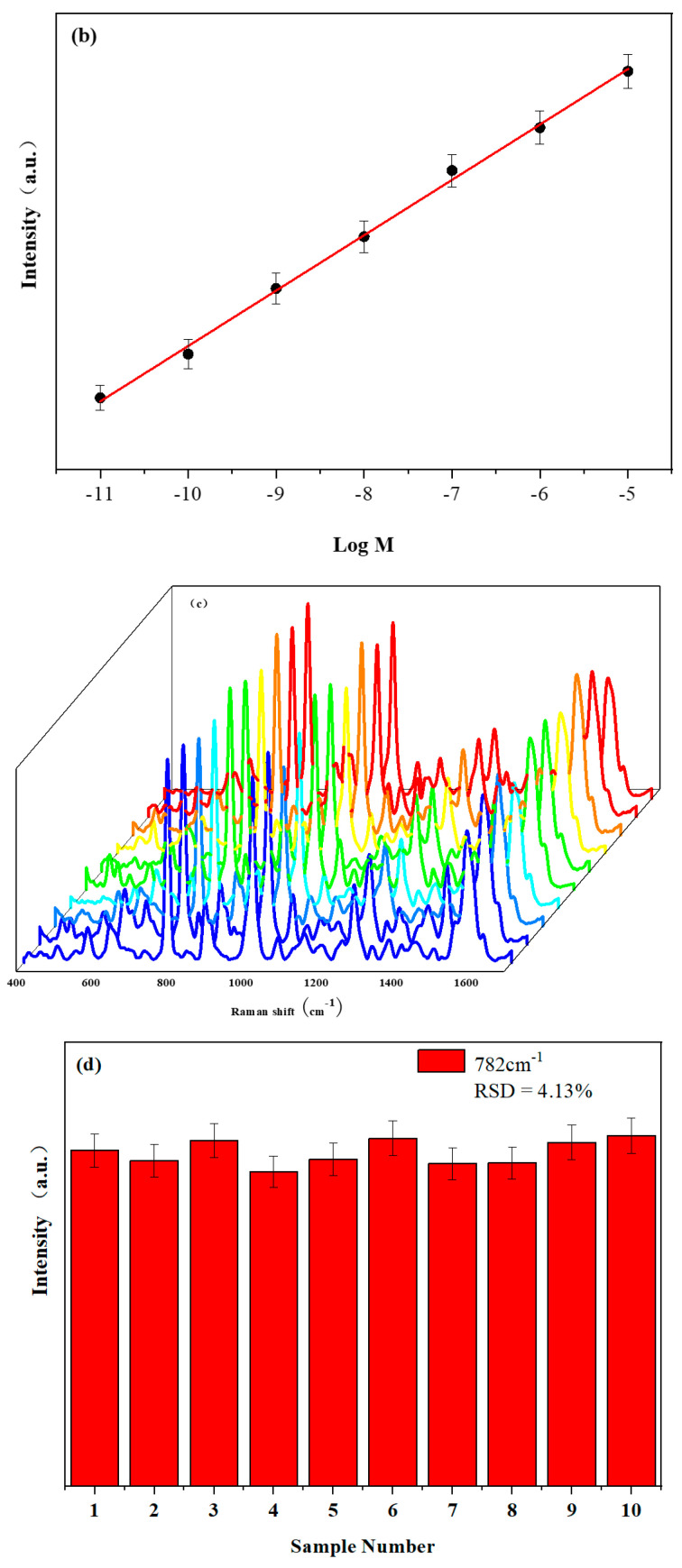
(**a**) SERS spectra for thiabendazole with different concentrations; (**b**) the relationship between peak intensity at 782 cm-1 and logarithmic concentration of thiabendazole; (**c**) SERS observations for 10 random points throughout the Z-shaped nanorods; (**d**) SERS intensity distribution and relative standard deviation of 782 cm^−1^ for 10 random points.

To get the polarization dependence of tilted silver nanorod arrays, a FDTD theoretical simulation is performed to show the distribution of hotspots in the nanostructures. The parallel light is placed 400 nanometers above the tilted nanorod array with a wavelength range of 300–800 nm. Figure 6a shows the geometric model of the inclined silver nanorod array and the inclination angle between the silver nanorod and the substrate plane is 45°, and the 5 × 5 nanorod array is set as the minimum periodic unit in the periodic array. The yellow and purple cross-sectional areas are the locations where the electromagnetic field monitor is placed. Through the simulation of the Y–Z plane, the hotspots in the tilted silver nanorod array mainly exist in the gaps between the nanorods. Different from the usual planar two-dimensional SERS substrates, tilted silver nanorod arrays also exhibit strong electromagnetic coupling in three-dimensional space. In the simulation diagram of the X–Y plane, a significant electromagnetic enhancement effect is observed in the middle region of the nano array due to the strong electromagnetic coupling between the neighbor nanorods. Figure 6b shows the geometric modeling of the V-shaped silver nanorod array with the inclination angle 45°, and the inclination angle for the second section of silver nanorod at 40°. The 5 × 5 V-shaped nanorod array is set as the smallest periodic unit in the periodic array, and the yellow, green, and purple cross-sectional areas are the positions where the electromagnetic field monitor is placed. In the simulation diagram of the Y–Z plane of the V-shaped nanorod array, hotspots not only exist in the gaps between the nanorods, but also have strong electromagnetic coupling at the tips of the V-shaped nanorods. The hotspots at the tips of the nanorods are caused by the tip effect. Compared with the simulation diagram of the Y–Z plane of the tilted nanorod array, the V-shaped nanorod array not only has more hotspot areas than the tilted nanorod array, but also has a higher intensity of electromagnetic coupling compared to the tilted nanorod array. Figure 6c shows the geometric modeling of the Z-shaped silver nanorod array built in the time-domain finite difference simulation software, with the inclination angle for the second section silver nanorod 45°, the second section of silver nanorod 40°, and the third section of silver nanorod 35°. In comparison to the tilted silver nanorods and the V-shaped nanorods, the Z-shaped nanorods show more hotspots and stronger electromagnetic coupling. Therefore, the Z-shaped nanorod array is chosen as a SERS substrate to detect the thiadiazole, as shown in Figure 7. As the thiadiazole concentration decreases, the detectable Raman signal intensity gradually decreases, and the SERS peak of thiadiazole can be successfully detected even at a concentration of 10^−11^ M. The energy band at 1580 cm^−1^ in Raman spectroscopy is due to the C-C stretching of thiadiazole. The moderate strength bands at 1275 cm^−1^ and 1456 cm^−1^ are attributed to C-C stretching and C-H bending, respectively. The Raman peak at 782 cm^−1^ belongs to the S-C stretching mode. The change in SERS peak intensity at 782 cm^−1^ is perfectly linearly correlated with the logarithm of thiadiazole concentration. The function between the characteristic peak intensity at 782 cm^−1^ and the logarithmic concentration of the extraction solution is obtained, y = 19,487 logx + 29,216, where R^2^ = 0.983, as shown in Figure 7b. When the logarithmic concentration is less than 10^−11^ M, the average peak intensity at 782 cm^−1^ deviates from linearity. The lowest concentration that can be determined to be statistically different from the blank is considered as the detection limit of the sensor, indicating the detection limit for thiadiazole is as low as 10^−11^ M. In practical applications, in addition to requiring a high sensitivity of SERS substrates, high uniformity is also required. Figure 7c shows almost identical Raman signals from 10 random points throughout the sample. To demonstrate the uniformity of the Z-shaped nanorods better, the intensity of the characteristic peak at 782 cm^−1^ is selected as a histogram in Figure 7d, and the relative standard deviation of 10 points was calculated to be 4.13%, confirming the excellent uniformity.

## 3. Materials and Methods

### 3.1. Materials

Ag (99.999%) targets were purchased from Beijing, China, TIANQI Advanced Materials Co., Ltd. (HZTQ). The 4-mercaptobenzoic acid (4-MBA) was bought from Sigma Aldrich Co., Ltd., St. Louis, MO, USA. Thiabendazole was bought from Thermo Scientific Co., Ltd., Waltham, MA, USA. The ultrapure water 18.2 MΩ was used.

### 3.2. Methods

To obtain a silicon column substrate after plasma etching, the silicon wafer is washed clean with a mixed solution of ammonia water, hydrogen peroxide, and deionized water (ammonia water: hydrogen peroxide: deionized water = 1:1:5). CF_4_ flux with 50 sccm is chosen for Si etching, Ar flux is 50 sccm, working pressure is 20 Pa, background pressure is 1 × 10^−3^ Pa, and etching power is 150 W. The regular arranged nanorods are immersed in 10^−3^ mol/L 4-MBA ethanol solution for 1 h and then the sample is rinsed with anhydrous ethanol to remove excess probe molecules that are free or physically adsorbed on the structural surface. Finally, the substrates are dried softly with N_2_ gas. SERS substrates are immersed in the thiabendazole solution with a concentration of 10^−6^ M, 10^−7^ M, 10^−8^ M, and 10^−9^ M, and then the substrates are dried softly with N_2_ gas. SERS measurements are excited by a 785 nm laser. SEM is performed on a far-field emission scanning electron microscope (15 kV, JEOL 7800F, Tokyo, Japan). SERS spectra are measured by Raman system with 785 laser irradiations (Ruhai, China). The magnetron sputtering system is FJL-700 (Shenkeyi, China).

### 3.3. FDTD Simulation

The electromagnetic field distribution in the prepared nanostructure is simulated by FDTD solution. The calculation in FDTD solution is based on Maxwell equation. The algorithm of the FDTD has the advantages of wide application range and high precision. The regular arranged nanorods are arranged on the X–Y plane and perpendicular to the X–Z plane. The dielectric constant and permeability values of Ag given by FDTD solution are used to simulate.

## 4. Conclusions

In this work, the inclined silver nanorod arrays, V-shaped nanorod arrays and Z-shaped nanorod arrays were grown on a dense silicon column substrate by magnetron sputtering. The electromagnetic enhancement performance of the SERS substrate was detected using a Raman spectrometer, and the hotspot distribution in three structures was simulated using FDTD solution. Based on the experimental results and FDTD solution simulation results, it was found that the Z-shaped nanorods showed excellent SERS properties with 3D hotspot distribution. The Z-shaped nanorod was applied for the detection of the pesticide thiabendazole with a detection limit as low as 10^−11^ M, indicating that it is a simple and efficient detection tool for the detection of the pesticide thiabendazole.
